# Quantifying effects of inattention on serial dependence in numerosity and orientation judgments using a Bayesian integration approach

**DOI:** 10.1186/s12915-026-02772-8

**Published:** 2026-10-31

**Authors:** Lena Silke Schädlich, Árni Kristjánsson, Claus-Christian Carbon, Alexander Pastukhov

**Affiliations:** 1 Department of General Psychology and Methodology, University of Bamberg, Bamberg, Germany; 2 Research Group EPÆG (Ergonomics, Psychological Æsthetics, Gestalt), Bamberg, Germany; 3 Department of Psychology, School of Health Sciences, University of Iceland, Reykjavík, Iceland

**Keywords:** Serial dependence, Attention, Visual perception, Computational modeling, Bayesian statistics

## Abstract

**Background:**

Our perceptual judgments are influenced by recent experience. In three experiments, we examined how such *serial dependence* in numerosity and orientation judgments is influenced by attention. Participants estimated numerosity in dot clouds (Experiment 1) or the orientation of Gabor patches (Experiment 2: high contrast, Experiment 3: low contrast). These tasks were paired with an attention-demanding central task to modulate attention in a dual-task design.

**Results:**

We found stronger serial dependence under divided attention across all experiments. Furthermore, we employed an ensemble of Bayesian models to disentangle the influence of attention, sensory evidence, and prior responses in numerosity and orientation judgments. Our six models differed in which of the three components of prior experience – average sensory evidence, prior sensory evidence and prior response – were included and how they expressed the influence of history on perceptual judgments. We found that all three factors influence perceptual judgments with attractive effects for prior responses and *average* sensory evidence, but a repulsive effect for prior sensory evidence. Importantly, the strength of the influence on individual components depended on the nature of the task (numerosity or orientation judgment), the quality of sensory evidence, and availability of attention.

**Conclusions:**

Our findings suggest that perceptual decisions arise from a context-specific integration of prior experiences. This means that the magnitude and even the sign of serial dependence, whether it is overall attractive or repulsive, reflect specific combinations of factors (task, quality of evidence, attention, etc.) and highlight the importance of computational modeling for disentangling the individual effects.

**Supplementary Information:**

The online version contains supplementary material available at https://doi.org/10.1186/s12915-026-02772-8.

## Background

Visual perception always occurs within a temporal context. In a phenomenon called *serial dependence,* perceptual decisions about the stimuli around us are biased by previous perceptions [1–5]. Such *serial dependence* applies to all kinds of perceptual decisions ranging from judgments about very basic stimulus properties like the orientation of Gabor patches [6] to higher-level judgments about facial expressions or facial identity [2, 7] and emotion perception [8]. Serial dependence is usually considered to be an adaptive phenomenon that ensures perceptual continuity based on the assumption that the world around us is generally stable [3, 9–11]. It can be either attractive, manifesting as a bias in perceptual decisions *toward* a particular value (numerosity or orientation) or repulsive, producing a bias *away* from it.

But other fundamental constraints on cognition limit how much information can be processed at any given moment. We cannot pay attention to all the visual input hitting our eyes as we walk across Times Square in Manhattan. Selective attention fundamentally affects what we see. We choose to attend to our partner walking with us, rather than the brightly colored advertisements on the screens around the square. In fact, diverting attention can result in stunning misses of seemingly highly pertinent stimuli [12–15]. Attention is thought to affect appearance [16], increase discriminability [17] and speed responding in perceptual tasks [18]. Notably, attention is also heavily dependent upon previous stimulus history – attentional priming strongly influences subsequent attention deployments – stimuli with previously attended characteristics tend to be found faster and discriminated more accurately than others [19–22]. Additionally, the converse can also occur; serial dependence can have a modulatory effect on attention [23–25].

Since both serial dependence and attention are strongly affected by temporal context, do these two effects interact, and if so, how? Fischer and Whitney [6] found that explicit instructions to attend led to stronger serial dependence. Fritsche and De Lange [26] found that when stimulus size was attended on a preceding trial, the effect of previous stimulus orientation on current judgments about orientation was smaller than when orientation was attended. Others have found that when observers perform tasks involving the orientation of a stimulus, and a secondary feature such as the pattern of the stimulus or its color is completely irrelevant, changes in the secondary feature have little influence on serial dependence [27–29]. However, when a secondary feature (color) is relevant to a primary motion discrimination task, serial dependence occurs for stimuli that share the secondary feature [30]. Houborg et al. [31] investigated whether distracting stimuli leave any trace on sequential judgments of line orientation. They found that distractors had little effect on attractive serial dependence but found that they caused repulsive serial dependence pushing perceptual decisions away from previous stimuli. Rafiei, Chetverikov, et al. [32] had observers search for an oddly oriented target line among distractor lines of various orientations around a mean value differing from the target orientation. Observers performed the search for a few trials in a row and then serial dependence was tested from either the target or the set of distractors. They found that distractors tended to cause repulsive serial dependence, while the targets caused attractive serial dependence, suggesting that the modulating effects of attention depended on the role that the stimuli played – whether they were targets to find, or distractors to ignore. Rafiei, Hansmann-Roth, et al. [24] argued that attractive biases pull the perception of target stimuli towards previous target features. Conversely, repulsive biases from distractors push perception of the target away from the distractors arguing that actively ignored information facilitates continuity in visual perception in addition to the previously documented serial dependence from target stimuli.

The coexistence of attractive and repulsive effects is also revealed by further differentiation of low-level adaptation in perception and perceptual decision-making: there is some evidence that past decisions evoke attractive serial dependence, while sensory processes seem to have a repulsive effect similar to visual aftereffects and adaptation [33–35]. Supporting this idea, Sadil et al. [36] found through two-factor modeling that for orientation judgments, serial dependence can be understood as the integration of both attractive biases towards the prior response and repulsive biases from prior evidence. However, Cicchini et al. [37] challenged this interpretation, arguing that attractive serial dependence during passive viewing or simple detection tasks – without any explicit judgment of stimulus properties – indicates that attractive priors operate at lower levels of perceptual processing.

The interaction between repulsive and attractive effects may also depend on how the temporal context is utilized to predict future perception. A powerful demonstration of the importance of presentation order comes from Pascucci and Plomp [38]. They tested serial dependence under conditions that induced so-called *representational momentum* [39]. A sequence of oriented Gabor patches unfolded either randomly or following rotational momentum. They found that serial dependence was modulated by the “expected” orientation in the display. Pascucci and Plomp [38] interpreted this as indicating that perceptual decisions are influenced by both past and future states of the visual input. A related finding was reported by Blondé et al. [33], who varied the temporal correlations in the features of a Gabor patch at two timescales, within and across trials. They found that increasing the temporal correlation in the stimulus history led to repulsive serial dependence, while higher temporal correlation in decision history reduced attractive serial dependence.

Orientation judgments have extensively been used for studying visual serial dependence (see [2]). Adjustment tasks are often used, where participants are asked to reproduce the perceived orientation of briefly presented and randomly oriented Gabor patches by adjusting a response bar [6, 9, 28, 29, 38]. Overall, mostly attractive effects have been reported with stronger serial dependence for more similar prior orientations [2].

Another example of serial dependence comes from studies where participants make judgments about numerosity, such as how many dots there are in a dot array [40–42]. Cicchini et al. [40] reported that apparent logarithmic representations of numbers could reflect linear representations when previous decisions were taken into account (see Dotan and Dehaene [43]). Cicchini et al. asked their observers to judge the numerosity of clouds of black and white dots on a grey background. They found a strong relationship between the mapping of the dots onto a number line on the current trial and the number of dots on the previous trial, in other words, serial dependence akin to what was previously seen for judgments of orientation [6]. They also found that this effect was modulated by attention: When observers performed a difficult search task, that was concurrently presented with the number task on a different part of the screen, this serial dependence in numerosity judgments was stronger.

### Current aims

Our aim here was to assess the relationship between attention and serial dependence in numerosity and orientation judgments. Although previous experiments have demonstrated various interactions between these two factors, the nature of their relationship remains unresolved. Therefore, we used modeling to estimate how the independent contribution of different factors could provide crucial insights into this interaction. To this end, we conducted three experiments examining how participants estimated numerosity in dot clouds (Experiment 1) or the orientation of Gabor patches (Experiment 2: high contrast, Experiment 3: low contrast) under different loads from a concurrent attention-demanding central task. We selected these two tasks because of their markedly different nature, which allowed us to assess commonalities and differences in serial dependence and their interaction with attention across distinct domains of visual perception. We focused on three measures of prior perceptual experience as predictors of perceptual decisions: 1) average sensory evidence, accumulated over the course of the entire experiment (up to the trial in question), 2) prior sensory evidence, i.e., the stimulus on the preceding trial, 3) prior response, i.e., the response on the preceding trial. These three components characterize the preceding history on both long-term (average sensory evidence) and short-term (prior sensory evidence and prior response) time scales. Note that we did not include the average response (long-time scale decision history) as, unlike its short-time scale counterpart, it is very similar to average sensory evidence and any deviations tend to average out.

In our modeling, we employed an ensemble of Bayesian models that differed in which of these three components of prior experience were incorporated and how they expressed any influences of history on the perceptual judgment. Below we demonstrate that our computational modeling suggests task- and attention-specific integration of these prior experiences. More generally, this indicates that no general predictions about the magnitude or the sign of serial dependence influences can be generated without taking these multidimensional influences of attention, sensory evidence and prior responses into account.

## Results

We investigated how perceptual decisions are affected by an interaction between attention, sensory evidence (both prior and current) and prior responses. To this end, the participants performed numerosity (Experiment 1) and orientation (Experiments 2 and 3) judgment tasks under full attention (single task) and divided attention (dual task) conditions. Next, we quantified the results using a model that predicts perceptual decisions using sensory evidence for current and previous trials as well as average sensory evidence and prior response (*Average Evidence, Prior Evidence, and Response integration model*). While this was not the only model we tested (see Methods section and repository for details on models), our best-fitting model indicated that all three components – average sensory evidence, prior sensory evidence, and prior response – must be incorporated in a model of perceptual decision-making in these tasks to best explain the data.

**Fig. 1:**
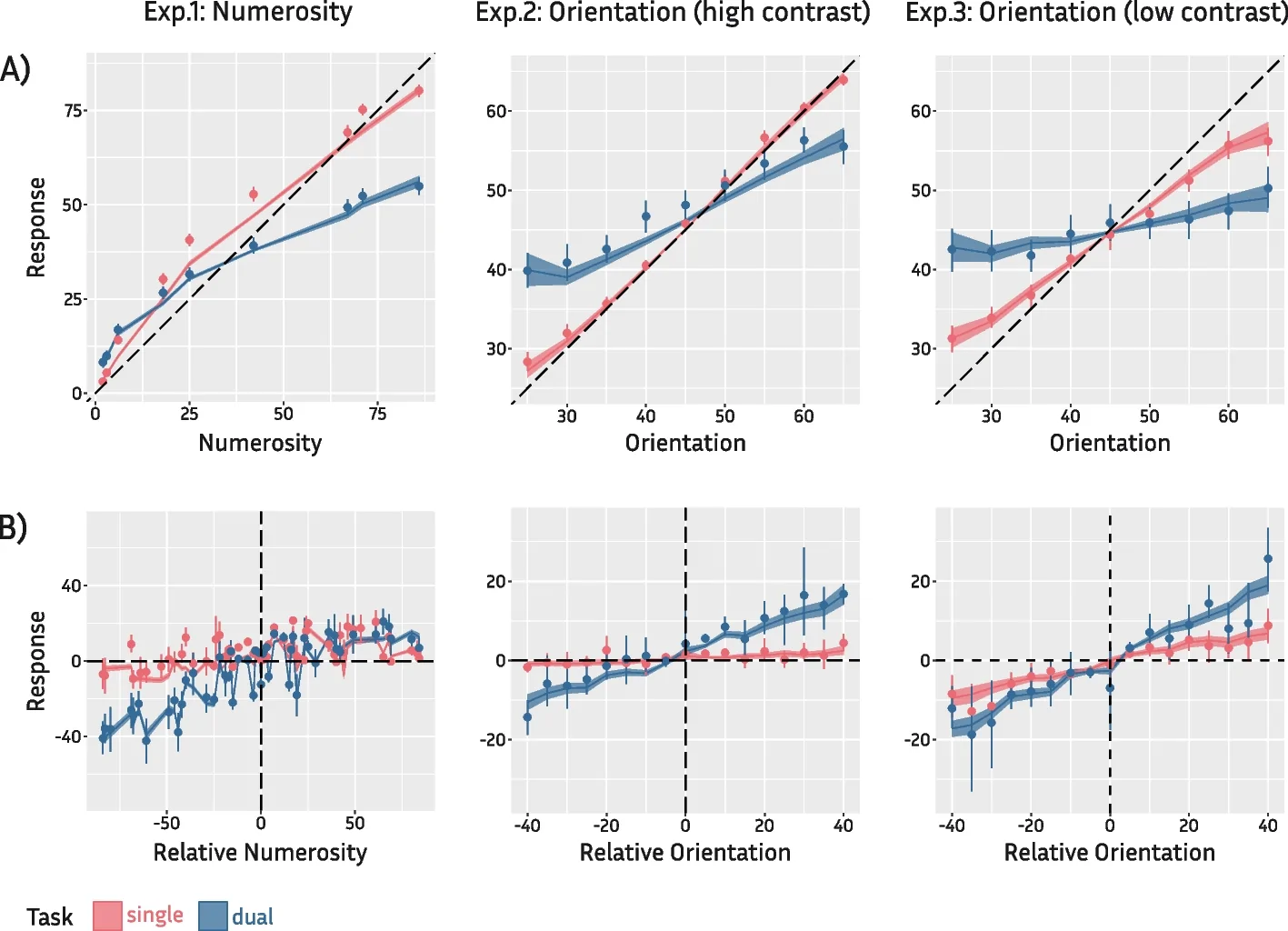
Behavioral results versus model predictions. Circles and error bars correspond to the mean and 97% non-parametric bootstrapped confidence intervals for group averages. Stripes depict 97% credible intervals for group average posterior predictions. The top row (**A**) illustrates absolute effects with stimulus magnitudes (numerosity or orientation) on the x-axis and responses on the y-axis. The bottom row (**B**) shows relative effects with the difference in stimulus to the previous trial on the x-axis and the relative response (difference in response to presented stimulus) on the y-axis. In all plots, the results are grouped for the single-task (red) and dual-task conditions (blue)

The behavioral results and the posterior predictions for the *Average Evidence, Prior Evidence, and Response integration model* are summarized in Fig. 1. Figure 1A shows behavioral results (circles and error bars) and posterior predictions (stripe) for each stimulus level. Overall, the model captures the shape of the response curve and the difference between the two attention conditions across all experiments. Figure 1B illustrates the effects relative to the stimulus on the previous trial. For the divided attention (dual task) conditions, there was clear serial dependence in all experiments. For the full attention (single task) condition, it is evident for Experiment 1 (numerosity judgment) and Experiment 3 (orientation judgment, low contrast), but there is little systematic bias towards prior evidence in Experiment 2 (orientation judgment, high contrast).

For our modeling, we assumed that prior response, prior sensory evidence, and average sensory evidence bias perception, either attracting or repulsing the perceptual decision. All three memory sources were modulated by their relevance to the current judgment controlled through a Gaussian distance measure and uncertainty. Similarly, all memories could have both attractive (positive estimates for the memory weight) or repulsive (negative values of the memory weight) effects. For both attractive and repulsive effects, larger absolute values indicate a larger influence on perceptual decisions, while zero weights indicate that a memory source is irrelevant to the perceptual decision.

**Fig. 2:**
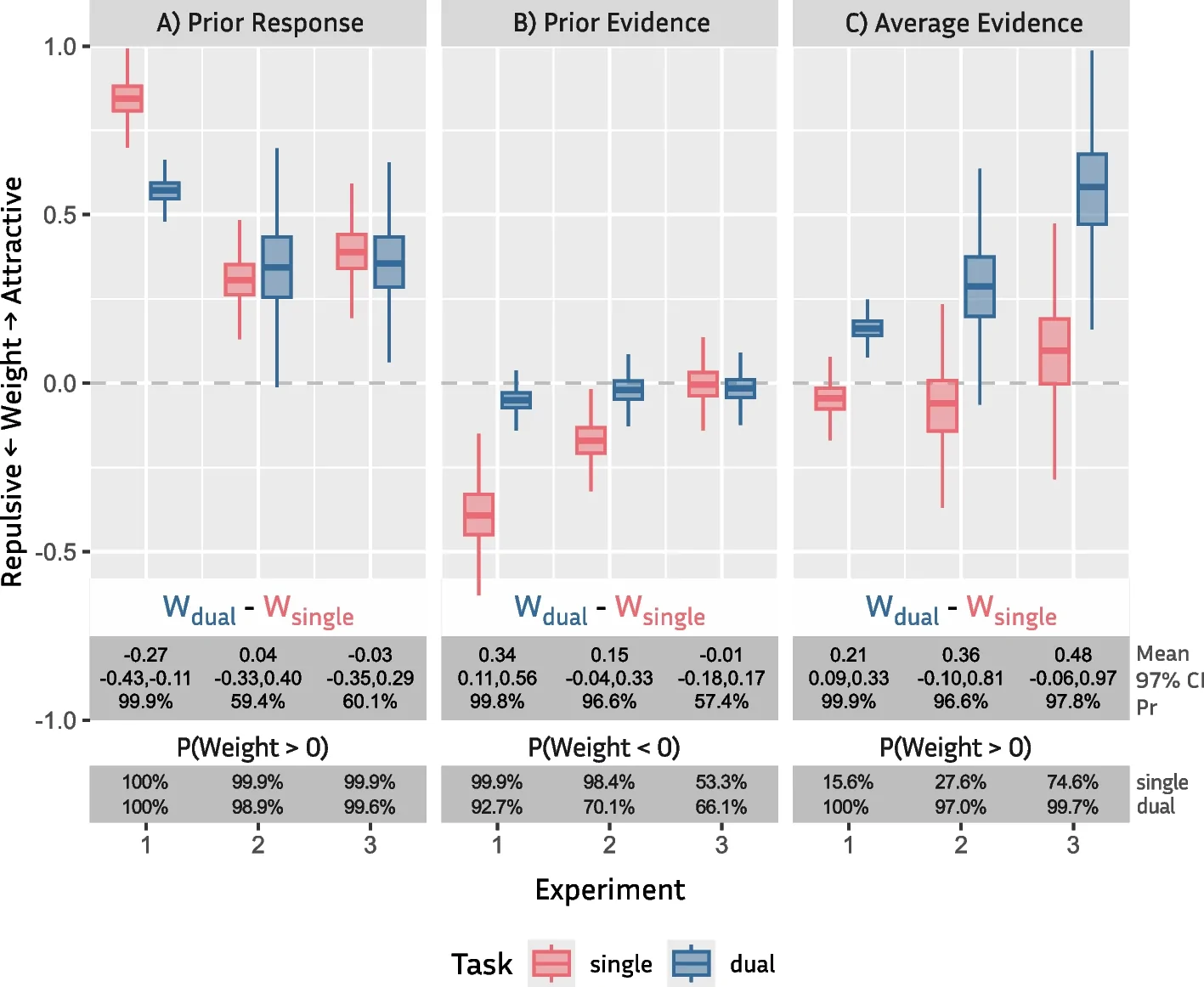
Posterior distributions of weights assigned to prior information for the three experiments. Values of the weights are shown on the y-axis. Estimates are grouped per attention condition (single task: red/left; dual task: blue/right). The columns show the weight of **A** prior response, **B** prior evidence, and **C** average evidence, respectively. The inset table shows differences between the two attention conditions (mean and 97% credible interval) and the probability of estimates being higher (positive values in the mean difference) or lower (negative values in the mean difference) in the dual-task than the single-task condition, and below the proportion of data above/below zero for each weight per experiment and attention condition. Please note that, in the poor attention (dual task) condition, both prior response and average evidence have an attractive effect, making precise estimation of the corresponding weights problematic, as the model can only account for the total attractive influence of both variables (note the bigger uncertainty about weight estimates for blue boxplots in subplots A and C)

To summarize the effects of the three memory types, firstly, the prior response had a consistent attractive effect (Fig. 2A, single task: *P*(Wr > 0) = 100%, 99.9%, and 99.9% for Experiments 1, 2, and 3, dual task: *P*(Wr > 0) = 100%, 98.9%, and 99.6% for Experiments 1, 2, and 3). The attractive effect was particularly strong for numerosity judgments but was weaker in the dual-task condition. For the orientation judgment task, a moderate attractive effect was not influenced by the attentional manipulation. In contrast, the influence of sensory evidence depended on both attention and the quality of the evidence. Specifically, prior evidence (the stimulus on the previous trial) had a repulsive effect on the perceptual decision but only in the full attention (single task) condition and for clear sensory evidence (numerosity stimulus in Experiment 1 and high contrast target in orientation judgment in Experiment 2), see Fig. 2B (single task: *P*(We < 0) = 99.8%, 98.4%, and 53.3% for Experiments 1, 2, and 3, dual task: *P*(We < 0) = 85.9%, 70.1%, and 66.1% for Experiments 1, 2, and 3). Conversely, average evidence had an attractive effect but only in the poor attention (dual task) conditions; see Fig. 2C (single task: *P*(Wct > 0) = 22.5%, 27.6%, and 74.6% for Experiments 1, 2, and 3, dual task: *P*(Wct > 0) = 99.9%, 97.0%, and 99.7% for Experiments 1, 2, and 3). When it comes to which memory effect had a stronger influence on perceptual decisions, the prior response effect appears to be the strongest, as it always had a larger or at least a similar influence compared to either sensory evidence memories. In short, our results suggest at least three different and, possibly, independent mechanisms that work at both sensory evidence and decision variable levels.

**Fig. 3:**
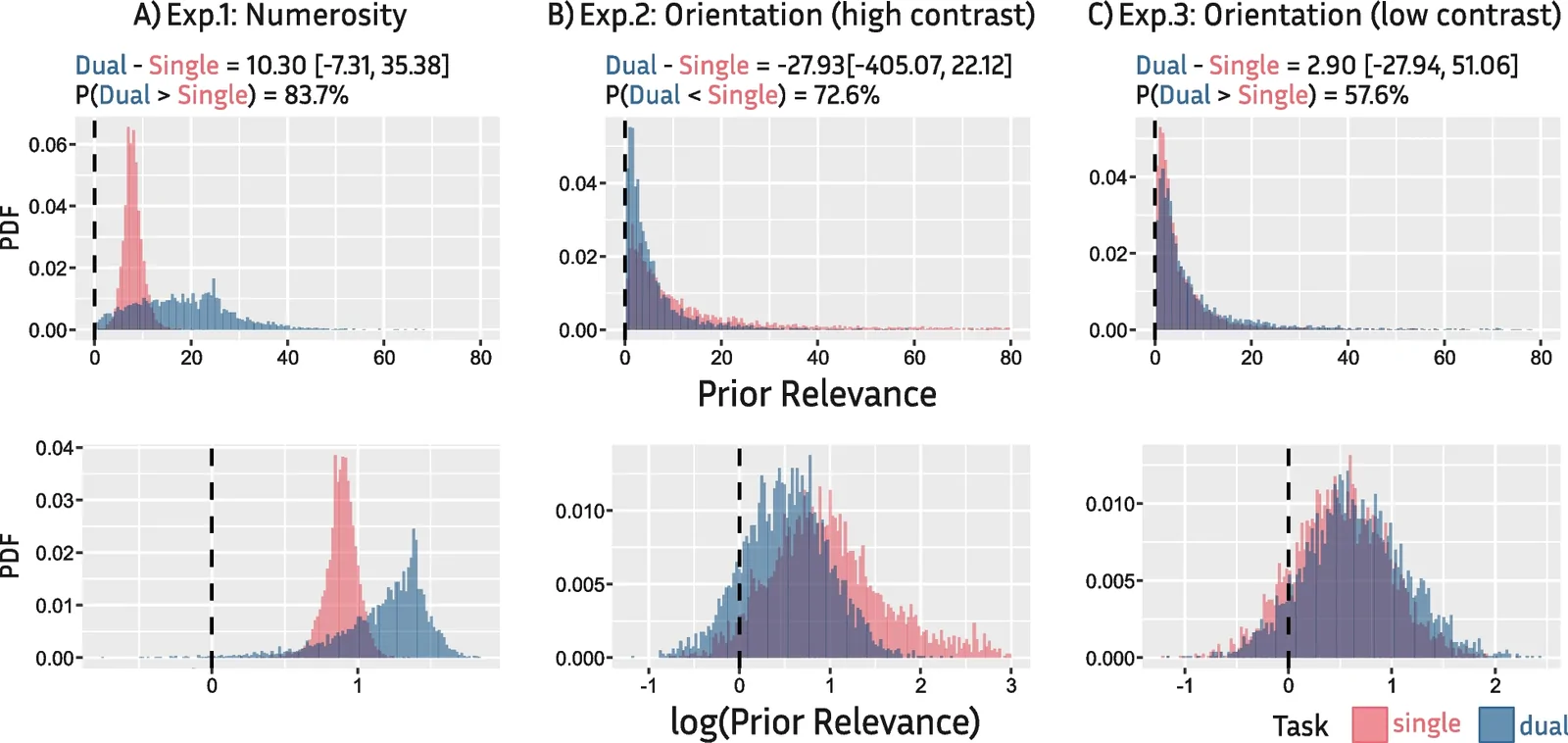
Posterior distributions for the relevance of prior information. Values of the parameter estimates on the x-axis with the probability density function (PDF) on the y-axis. Estimates are grouped per attention condition (single task: red; dual task: blue). The text above each plot shows the difference between the two attention conditions (mean and 97% credible interval) and the probability of estimates being higher or lower in the dual-task than the single-task condition. The columns divide results per experiment: **A** Experiment 1, **B** Experiment 2, **C** Experiment 3. The bottom row shows the same plots but with the x-axis transformed to the log10 scale

The influence of all three sources of information was modulated by their relevance to the decision. We quantified this via the Gaussian distance between prior information and current sensory evidence with parameter λ controlling how selective the model was. Specifically, smaller values of λ lead to higher selectivity, so that only values nearby the current evidence influence the decision. Conversely, larger values of λ mean that even values far away from the current stimulus can nonetheless bias the perceptual decision. For numerosity judgments (Experiment 1), we found lower selectivity in the dual-task condition (Fig. 3A). This indicates a stronger overall influence of prior information, as all three sources of information would be incorporated despite not being highly relevant for the judgment. In contrast, attention had no effect on prior information selectivity in orientation judgments (Fig. 3BC) and selectivity was not affected by stimulus contrast, as there was no difference in λ for Experiments 2 and 3 (single task: *P*(Exp.2 > Exp.3) = 70.6%, dual task: *P*(Exp.2 < Exp.3) = 57.0%).

**Fig. 4:**
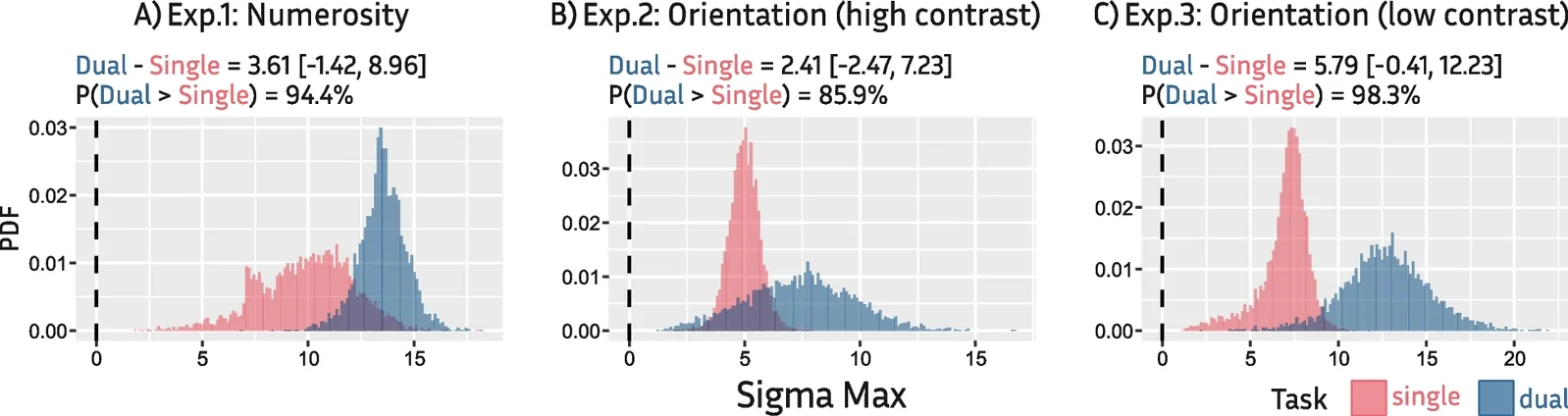
Posterior distributions for the maximal standard deviation of residuals. Values of the parameter estimates on the x-axis with the probability density function (PDF) on the y-axis. Estimates are grouped per attention condition (single task: red; dual task: blue). The text above each plot shows the difference between the two attention conditions (mean and 97% credible interval) and the probability of estimates being higher or lower in the dual-task than the single-task condition. The columns divide results per experiment: **A** Experiment 1, **B** Experiment 2, **C** Experiment 3

Finally, our model contained a term that quantified additional variability of responses that was not accounted for by other factors: the standard deviation of residuals. We found that for all three experiments, the poor attention (dual task) condition led to a higher variability of responses (Fig. 4). Similarly, we observed a higher variability of responses for lower contrast for the orientation judgment task (Experiment 2 versus Experiment 3: single task: *P*(Exp.3 > Exp.2) = 89.8%, dual task: *P*(Exp.3 > Exp.2) = 94.6%).

In the analyses, we opted to focus on the key comparison between different history traces and omitted presentation order effects. However, we found that the presentation order does not systematically modify serial dependence (see Additional file 1: Fig. S1). Furthermore, the resampled weights for only random order blocks show no difference in the overall pattern of prior effects (see Additional file 1: Fig. S2).

To summarize, we characterized participants’ responses using a model that included effects of average sensory evidence (sensory evidence accumulated over the course of the entire experiment up to the trial), prior sensory evidence, and prior response. For both numerosity and orientation judgments, poor attention led to a stronger influence of average sensory evidence and more variable responses. For both numerosity and orientation judgments, prior evidence had a repulsive effect and prior response and average evidence had attractive effects on the perceptual decision. Finally, the effect of prior response was strongest for all experiments and conditions.

## Discussion

We investigated the effects of prior experience and attention on perceptual judgments in numerosity and orientation tasks. We quantified participants’ responses using an ensemble of Bayesian models, which differed in how prior sensory evidence and prior responses were integrated into the current response. Our best-performing model relied on all three sources of prior information – the average sensory evidence, prior sensory evidence, and prior response – indicating that all three components have an influence on perceptual decisions. However, their strength depended both on stimulus-strength and attention. Specifically, an attractive effect of the prior response was present for all stimuli and attention conditions. In contrast, the repulsive effect of prior sensory evidence was seen primarily for the full attention condition with high-strength stimuli, whereas the attractive effect of the average sensory evidence appeared in the poor attention condition. In short, our results indicate that perceptual decisions are affected by a context-specific combination of various facets of prior experience where some, but not all, are affected by attention. Our results pose several questions, the main one being, how should we think about serial dependence?

### What is serial dependence?

The influence of prior perceptual judgments on the perception of a given trial is referred to as *serial dependence* [2, 3, 37]. As discussed in the introduction, the literature indicates that serial dependence exerts influences at various levels of perceptual processing [3] and seems to be a general characteristic of such processing. However, our results pose a question: Which specific component of prior information should we refer to using the term *serial dependence*? This choice would define the expected sign of the effect, as immediate prior sensory evidence has a repulsive effect whereas the prior response has an attractive effect. These contrasting effects align with what has previously been shown for orientation and motion judgments [34–36, 44]. Moon and Kwon [44] proposed that these two opposing biases are integrated additively as an optimization strategy.

Similarly, this would influence the answer to the question that we originally posed: How does attention influence serial dependence? As with the sign of the effect, the answer depends on which component we focus on. The influence of prior response appears to be little affected by full versus divided attention, whereas prior sensory evidence had an effect only under full attention. In short, we consider serial dependence as a useful umbrella term to signify an influence of overall prior experience on the current judgment. We think it is important to understand it in this broad way, when considering the effects of specific components of serial dependence in different studies. Rather than thinking of serial dependence as a single mechanism, it might better be thought of as a general characteristic of the neural processing of perceptual input.

### Does average prior evidence contribute to serial dependence?

We found that the average prior evidence influences perceptual decisions alongside immediate prior evidence and the prior response, which have separate influences on current responses [35, 36, 44]. The former effect can also be referred to as *regression to the mean* or *central tendency* [37, 45, 46] and is generally viewed as an effect separate from serial dependence to highlight the more immediate nature of the latter. Yet, the mechanisms responsible for influence of average prior evidence rely on the same information and differ only in the integration time scale. Whereas only the immediate past is considered for serial dependence, average evidence integrates over the entire prior perceptual experience. Accordingly, it may be more productive to view both serial dependence and regression to the mean as parts of a broad mechanism that integrates information over multiple time scales, rather than as independent processes.

In our models, we have distinguished the two by using a perfect integrator for the average prior evidence and a single trial long memory for sensory evidence. However, we must note that these are design decisions rather than any ground truth. It is likely that the influence of average prior evidence relies on a slow time scale, but a leaky rather than a perfect integrator. Similarly, it is equally possible that the memory of immediate sensory evidence is not so immediate. In the future, it would be prudent to estimate these factors by looking at commonalities or differences across different stimuli and paradigms.

### Attention influences how prior information is incorporated into perceptual decisions

Multiple studies have investigated the effect of attention on serial dependence. There are methodological differences in the manipulation of attention, as it can be modulated through pre-cueing, feature-based task relevance, the absence of explicit judgments and responses in some trials [2, 3, 37] or by using a dual task where participants are asked to perform a simultaneously presented task before responding to the primary task [40, 47]. The results of previous studies are mixed. Whereas some studies have shown that limited attention decreases serial dependence [2], other studies [40, 47], with memory load manipulations [48], report increased serial dependence. In addition, Pascucci et al. [3] argued that attention may primarily influence the direction of biases, with attention being necessary for attractive serial dependence.

Our results help us understand these seemingly contradicting findings by demonstrating a different attentional modulation of individual memory mechanisms and different overall contribution of these memories depending on the task and the reliability of sensory evidence. If we define serial dependence as a single bias that consists of the overall sum of competing attractive and repulsive influences, its overall change due to (in)attention will depend on which individual components were modulated the strongest and on the relative strength of attractive and repulsive influences. For example, in Experiment 3 (orientation judgment, low contrast) attention *decreased* overall serial dependence by decreasing the attractive influence of average sensory evidence (Fig. 2). In Experiment 1 (numerosity judgment), the overall attractive serial dependence was decreased slightly but only because an *increase* in the attractive bias of a prior response was offset by an *increase* in a repulsive bias of prior evidence and a *decrease* in the influence of average sensory evidence. Finally, in Experiment 2 (orientation judgment, high contrast) the overall decrease in attractive serial dependence was produced by an interplay between stronger repulsive effects of prior evidence and weaker attractive pull of average prior evidence. In all cases, attention does not change the sign (attractive or repulsive) of the individual biases but appears to have different effects based on the time scale of the memory, upmodulating more recent information (prior evidence and response) and downmodulating older average prior evidence.

Our results highlight the importance of modeling individual memory components rather than the overall effect of serial dependence. This means that there can be no simple answer to a question of “What is the effect of attention on serial dependence?” The prime example is Experiment 1, where the *increase* of both attractive and repulsive influences of recent experience led to a net decrease of the attractive effect. Similarly, in Experiment 2 the overall decrease represents a combination of increase, decrease and no change. The question of what the overall effect of attention on serial dependence is becomes moot, as it depends on which specific factors were entered into the model.

### Limitations

While we attempted to align the numerosity and orientation judgments as closely as possible, the remaining inconsistencies may allow different interpretations of the comparison between both tasks. We employed a linear response interval for numerosity (where dot clouds were transformed onto a number line), while orientation judgments utilized a reproduction task through line adjustment. We accounted for these differences in response modalities within our modeling. Further, the magnitudes of the stimuli were not identical: the difference between the lowest and highest numerosities was greater than the difference between minimal and maximal rotation angles, which also led to different step sizes between stimulus magnitudes. While initially replicating the numerosity judgment task of Cicchini et al. [40], we also aligned our methods with typical orientation judgment experiments to enhance the comparability of our results with previous findings [3, 9, 29]. Nonetheless, it would be ideal to make both the response and stimuli in future experiments as similar as possible to disentangle the effects of presentation and internal processing. Finally, in the case of the orientation task, it is possible that the perceptual decision was also subject to a repulsive effect of the cardinal axes, biasing it towards a 45-degree orientation. This orientation is also the expected value for the average prior evidence and, therefore, it is impossible to distinguish their influence in our data (the two predictors are collinear). Hence, our inferences with respect to average prior evidence could also be applicable to the cardinal axis bias.

It would also be of interest to include multiple levels of attention modulation to enable comparisons that extend beyond full versus poor attention. Further, the strong regression towards the mean observed in the dual-task condition of the orientation tasks in experiments 2 and 3 could be amplified by the design of the dual task itself. Participants had to make judgments based on the color of diagonally aligned squares for the color discrimination task. Participants may have recalled an orientation of 45° before making their orientation judgment on each trial in the dual-task condition. This may partly explain differences in the influence of average evidence observed between orientation and numerosity judgments.

## Conclusions

To summarize, our results show that serial dependence involves an interaction between perceptual decision mechanisms and perceptual history integrated over both short-time scales, reflecting immediate experience, and long-time scales, reflecting regression to the mean. In our experiments, the influence of individual components depended on the nature of the task, specifically numerosity versus orientation judgments, the quality of sensory evidence, and the availability of attention. This suggests that the magnitude and even the sign of serial dependence (attractive or repulsive) and its modulation by attention reflect specific combinations of factors, highlighting the importance of computational modeling for quantifying experimental results.

## Methods

### Participants

Ten undergraduates of the University of Bamberg (10 female, age range 20–35) took part in Experiment 1, twelve (12 female, age range 19–51) in Experiment 2, and twelve (8 female, 4 male, age range 20–29) participated in Experiment 3. In the first experiment, we initially also tested twelve participants, but one data file could not be saved due to technical problems and one participant was excluded due to poor central task performance (P(correct) = 51.5%; see Additional file1: Fig. S3). All procedures were in accordance with the national ethical standards on human experimentation and with the Declaration of Helsinki of 1975, as revised in 2008. The study was in full accordance with the ethical guidelines of the University of Bamberg and was approved by an umbrella evaluation for psychophysical testing of the university ethics committee (Ethikrat) on August 18, 2017. Informed consent was obtained from all observers before each experimental session.

All participants had normal or corrected-to-normal vision and normal color vision, tested by standard tests in situ, and were naïve to the purpose of the study. For their participation, they received a credit within the framework of a mandatory module of research participation in accordance with the standards of the University of Bamberg.

### Apparatus

Stimuli were presented on an EIZO CG245W display with the size of the visible area 51.7 cm × 32.3 cm, resolution 1,920 × 1,200 pixels, and refresh rate 60 Hz. At the viewing distance of 65 cm, a single pixel subtended 0.023° of visual angle. The head of the participants was stabilized by chin and forehead rests that ensured consistent viewing position and angle. Participants responded using a computer mouse (numerosity or orientation task) and the arrow keys of a computer keyboard (central task).

### Software

Stimuli were generated using custom code and the PsychoPy library [49]. Data analysis was performed in R [50] using Tidyverse library [51]. Models were programmed and sampled using the Stan probabilistic programming language [52]. A leave-one-out information criterion was computed using the “loo” library [53].

### Central task

**Fig. 5:**
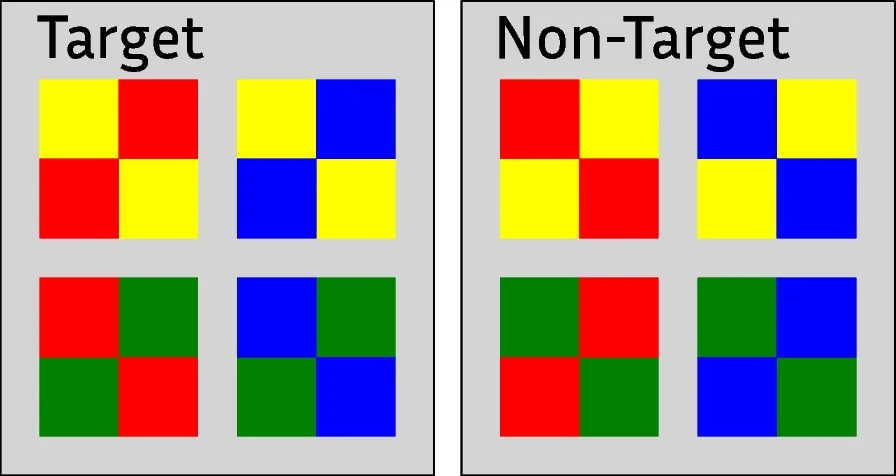
Target and distractor color combinations for the central task

We used the same attention-demanding central task throughout all experiments, following the design in Cicchini et al. [40]. The distractor stimulus consisted of four colored squares that covered the central 1° square. Their color combination could either represent a target (both top-left and bottom-right squares yellow or both top-right and bottom-left green) or a non-target (any other color combination), see Fig. 5.

The central task was presented in all trials of all experiments. The first block in every experiment was used to acquaint participants with the task and they had to respond on the central task only. In all other blocks, in the single-task condition participants were instructed to ignore the central task. In the dual-task condition participants had to divide attention between the two tasks and reported on the central task first.

### Experiment 1: numerosity judgments

**Fig. 6:**
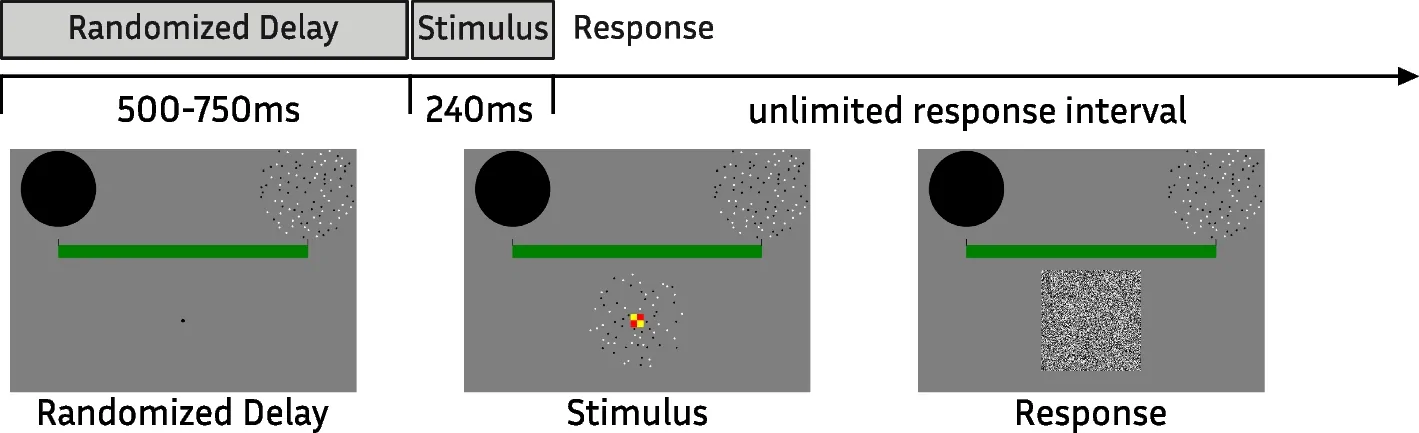
Experimental procedure and display for Experiment 1

The stimuli and procedure followed Cicchini et al. [40] except for an additional manipulation of presentation sequence order (see below). Each numerosity stimulus consisted of 2, 3, 6, 18, 25, 42, 67, 71, or 86 non-overlapping dots (half black and half white) at 90% contrast. The stimuli were displayed within a circle of 8° diameter, sparing the central 1° square for the central task (see above). The mask consisted of random black and white pixels covering an 8° square and it remained visible throughout the response interval. Participants reported the perceived numerosity using a green number line (20° long, 1° wide) that had example dot clouds on both extremes (one dot on the left and 100 dots on the right side, covering the circle of 3°) and was always present on the screen. On each trial, after a random onset delay (500–750 ms) both the numerosity and central task stimuli were presented for 240 ms, followed by a mask and an unlimited response interval (see Fig. 6). For the dual-task condition, participants responded first on the central task (using the left key for the *target* and the right key for *distractors*) and then on the numerosity task by clicking on the number line.

Each experimental session consisted of 62 blocks. The conditions for the first two blocks were fixed, starting with a single central task condition (see above) and the second block always involved a single numerosity task with random numerosity order. The remaining blocks consisted of ten repetitions of six conditions presented in random order: 2 attention conditions (single or dual task) × 3 presentation order conditions (ascending, starting at 2 and going up; random; and descending, starting at 86 and going down). Each block contained nine trials, so that each numerosity was presented only once.

### Experiment 2: orientation judgments (High contrast)

**Fig. 7:**
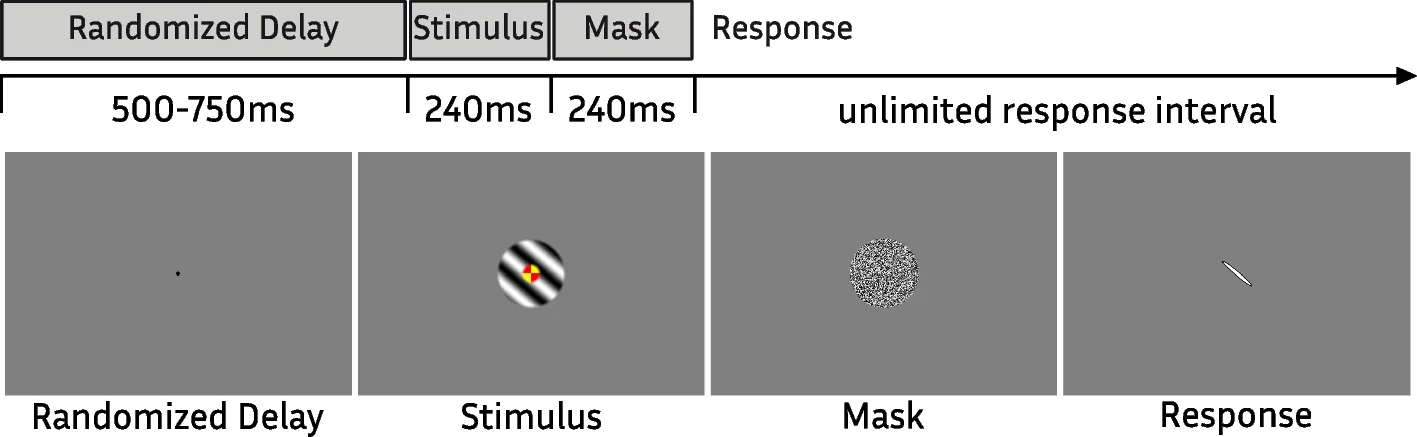
Experimental procedure and display of Experiment 2

Experiment 2 followed the same overall design and procedure as Experiment 1, but the numerosity judgment was replaced by an orientation judgment that followed a design of a widely used adjustment task [3]. Specifically, Gabor patches (diameter 3 degrees of visual angle, spatial frequency 3 cycles per degree, 100% contrast) were presented in nine varying orientations ranging from ± 25° to ± 65° in 5° steps. The four colored squares covered the central circle of 1° diameter. The procedure was similar to Experiment 1 (see Fig. 7) with each trial starting with a random offset delay (500–750 ms), followed by the stimulus presentation (240 ms), the random-noise mask that covered the central 8° (240 ms), and the response interval. During the response interval, the response widget appeared in a random orientation and participants adjusted it with the mouse scroll wheel to report the perceived orientation of the Gabor patch. The participants finalized their report via a left mouse click.

The 62 blocks of each experimental session were structured identically to Experiment 1 with the orientation either in random order or starting at ± 25° or ± 65° and changing clockwise or counterclockwise for the ascending and descending order condition, respectively. We transformed the negative orientation and corresponding responses to positive values before analysis.

### Experiment 3: orientation judgments (Low contrast)

To increase the task difficulty of the orientation judgment in Experiment 3, we decreased the contrast of the Gabor patches (2.5%). All remaining stimuli and procedural details were identical to those used in Experiment 2 (see above).

### Statistical methods

We computed statistics for all model parameters using the samples from the posterior distribution. For each parameter, we computed the mean and 97% credible interval (CI, also known as compatibility interval), a range containing 97% of the probability mass based on values from the sampled posterior distribution. We chose to use a 97% CI to emphasize that commonly used interval widths are conventional choices. This choice of a prime number is equally arbitrary and does not affect the conclusions.

To capture low-level perceptual effects such as serial dependence with high precision, we employed a dense within-participant design. Each participant completed 558 trials, yielding a dataset that allows for the estimation of factor-specific effects and the assessment of selective contributions to the observed bias. As the relevant variance in serial dependence paradigms is captured primarily within individuals across repeated observations, statistical power is driven primarily by the number of observations per condition rather than the absolute number of participants. The chosen sample size aligns with standard and well-established benchmarks in the serial dependence literature (e.g., [6, 34]). Population-level inferences were modeled using a Bayesian multi-level framework with regularizing priors. This approach employs partial pooling to balance within-subject precision with between-subject variance, enabling generalization to the wider population. Simultaneously, conservative, regularizing priors guard against overfitting and prevent the inflation of effect sizes, ensuring that population-level estimates remain robust and stable.

To understand which information sources are relevant for decision making, we compared the models using the leave-one-out cross-validation (LOO-CV) information criterion [53]. Please note that information criteria quantify predictive effectiveness of models but cannot validate their causal structure. Therefore, they cannot identify the “true” model but can be used as an indicator of which model is likely to correspond more closely to the generative process that produced the data and, thus, which predictors are likely to play a role.

### Statistical models

**Fig. 8:**
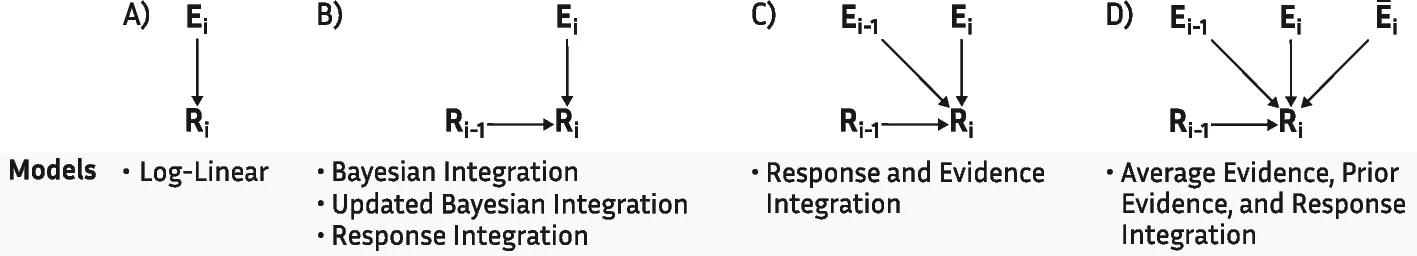
Directed-acyclic graphs for statistical models used in the study. *R*_*i*_ and *E*_*i*_ stand for the response and evidence on the current trial, *R*_*i-1*_ and *E*_*i-1*_ are response and evidence on the previous trial, and ${\overline{{{E}}} }_{{{i}}}$ is the average sensory evidence

To gain a deeper understanding of the underlying mechanisms of serial dependence, we used not only the original Bayesian integration model and the log-linear model employed by Cicchini et al. [40], but in addition, constructed a series of Bayesian integration models of varying complexity. The additional models were designed to allow for more flexibility as well as to facilitate parameter interpretations but differed in the way previous information (response and average and recent evidence) was integrated to estimate the current response (causal diagrams presented via directed-acyclic graphs for all models are presented in Fig. 8). We compared all models via LOO-CV information criterion (see Table 1) and for all three experiments the *Average Evidence, Prior Evidence, and Response integration model* performed the best. Therefore, in the Results section, we discuss only the *Average Evidence, Prior Evidence, and Response integration model*, but for the sake of completeness, we included descriptions of other models below and in the online repository [54].

**Table 1:** Statistical comparison of all models for each Experiment via LOO-CV: Log-linear model, Bayesian integration model, Updated Bayesian integration model, Response integration model, Response and Evidence integration model, Average Evidence, Prior Evidence, and Response integration model. *ΔELPD* the difference in expected log-predicted density relative to the best model (mean ± standard error). *Weight* the relative predictive weight for each model (sum up to 1)

Model	ΔELPD	Weight
*Exp.1: Numerosity*		
Average Evidence, Prior Evidence, and Response integration		0.728
Response and Evidence integration	−68.4 ± 15.5	0.068
Updated Bayesian integration	−238.3 ± 28.4	0.000
Response integration	−240.2 ± 28.2	0.000
Log-linear	−583.9 ± 67.8	0.189
Bayesian integration	−1436.6 ± 64.23	0.007
*Exp.2: Orientation (high contrast)*		
Average Evidence, Prior Evidence, and Response integration		0.827
Response and Evidence integration	−97 ± 20.1	0.032
Response integration	−122.7 ± 24	0.022
Updated Bayesian integration	−124.8 ± 24	0.000
Bayesian integration	−149.3 ± 24.9	0.119
*Exp.3: Orientation (low contrast)*		
Average Evidence, Prior Evidence, and Response integration		0.776
Response and Evidence integration	−168.4 ± 31	0.065
Updated Bayesian integration	−216.3 ± 37.3	0.144
Response integration	−218.1 ± 37.3	0.016
Bayesian integration	−13,373,059 ± 905,160.9	0.000

#### Log-linear model

The simplest model that we used was a log-linear model (see Fig. 8A) that predicts numerosity judgment as a weighted combination of the sensory evidence and its logarithm; see Cicchini et al. [40]. This model treats each trial as independent and, therefore, cannot capture dependence of the response on prior perceptual experience and decisions. Although the model provides a good fit to the data, it has limited explanatory power as it provides little guidance about the possible underlying perceptual mechanisms. Finally, this model is applicable only to numerosity and was therefore not used for orientation judgments in Experiments 2 and 3.

#### Bayesian integration model

The next model that we implemented was a *Bayesian integration model* presented in Cicchini et al. [40], see Fig. 8B. Here, the response for the current trial is computed as a weighted combination of the current sensory evidence and the response on the previous trial. The weight of the prior response reflects uncertainty about current and prior sensory evidence, as well as the relevance of prior response measured via Euclidean distance to the current sensory evidence.

#### Updated Bayesian integration model

Although the *Bayesian integration model* has the advantage that it explicitly links sensory evidence and prior decision, it provides a fairly poor fit to the data and not all of its parameters are directly interpretable. Therefore, we developed an *updated Bayesian integration model* that uses the same information (Fig. 8B). First, we modeled uncertainty about current numerosity via a Beta distribution allowing both monotonic and non-monotonic dependence. Second, we used an explicit parameter that controlled weight of the prior response for a given attention condition. Third, we computed relevance of prior response via Gaussian distance which has an explicit parameter to control it. Taken together, they allow for a more direct comparison of how attention availability influences all three components.

#### Response integration model

The limitation of the two Bayesian integration models above is that they allow only for an attractive effect of the prior response. In the *Response integration model,* we reparametrized the model allowing the weight of prior response to be both positive and negative and, thus, allowing for both attractive and repulsive effects. As the two Bayesian integration models above, it used the same inputs: current sensory evidence and prior response, Fig. 8B. Note that for weights of prior response within the range of 0 to 1, this model is equivalent to the one above.

#### Response and evidence integration model

Next, we surmised that it is possible that prior sensory evidence may also have an effect on perceptual decisions. Therefore, we created the *Response and Evidence integration model* that computed responses as a sum of the current sensory evidence, the weighted prior response, and the weighted prior sensory evidence (Fig. 8C). The weights of prior response and evidence were independent of each other and – similar to the implementation in the *Response integration model* – not restricted to a limited interval but allowed for both attractive (positive values) and repulsive (negative values) serial effects.

#### Average evidence, prior evidence, and response integration model

In all the models above, only the immediately preceding sensory evidence and response influenced the decision. However, sensory evidence can also be integrated over all trials to construct an estimate of the distribution of sensory signals. This information acts as another longer time-scale source of information during decision making, acting as a prior and causing regression to the mean. Therefore, we created the *Average Evidence, Prior Evidence, and Response integration model* that also includes the average sensory evidence, computed as an average across all preceding trials irrespective of the task, as a predictor (Fig. 8D). For this, we added a parameter that controls the weight for such influence of average evidence similarly to the weight of prior response and evidence. Please note that this does not imply identical neural mechanisms for all three history effects but expresses their influence in a consistent way, allowing us to directly compare both sign and magnitude of these effects.

We used covarying random effects for all model parameters sampled from a multivariate Gaussian distribution per task and participant. Hence, although we do not mention this in the description below for simplification, all parameters are both task- and participant-specific. We used weakly regularizing priors built upon the posterior predictions in previous models [40] and non-centered reparameterization using Cholesky factor decomposition for the variance–covariance matrix *S*. To simplify understanding and comparison in the main text, the full model definition can be found in the appendix (see Additional File 2: Full Model Definition for Average Evidence, Prior Evidence, and Response integration model).

Participants’ responses are estimated as a weighted sum of the current, average and prior sensory evidence and the response on the previous trial. The variables *E*_*i*_ and *E*_*i-1*_ refer to the sensory evidence (numerosity in Experiment 1, orientation in Experiment 2 and 3) on current and previous trials and ${\overline{E} }_{i}$ to average evidence up to the current trial. Variables *R*_*i*_ and *R*_*i-1*_ refer to responses on the current and previous trials.

1 $$R_i\sim\mathrm{Normal}\left(\mu_i,\sigma_i\right)$$

2 $${\mu}_{i}= \left\{\begin{array}{cc}{E}_{i}& i=1\\ {E}_{i} +{W}_{CT}\cdot \left({\overline{E} }_{i}-{E}_{i}\right)+ {W}_{{R}_{i-1}}\cdot \left({R}_{i-1}-{E}_{i}\right)+{W}_{{E}_{i-1}}\cdot \left({E}_{i-1}-{E}_{i}\right)& i>1\end{array}\right.$$

3 $${{\mathrm{W}}_\mathrm{R}}_{\mathrm{i}-1}=\mathrm{W}_\mathrm{R}^\mathrm{max}{\mathrm{U}}_\mathrm{i}e^{-\frac{\left(E_i-R_{i-1}\right)^2}{\lambda^2}}$$

4 $${{W}_{E}}_{i-1}={W}_{E}^\mathrm{max}{U}_{i}{e}^{- \frac{{\left({E}_{i}-{E}_{i-1}\right)}^{2} }{{\lambda }^{2}}}$$

5 $${W}_{CT}={W}_{CT}^\mathrm{max}{U}_{i}{e}^{- \frac{{\left({E}_{i}-{\overline{E} }_{i}\right)}^{2} }{{\lambda }^{2}}}$$

6 $${\sigma}_{i}= {\alpha}_{\sigma }+ {\beta}_{\sigma }{U}_{i}$$

7 $${\overline{E} }_{i}=\frac{{\sum E}_{i}}{{N}_{i}}$$

The weights for the three memories are a function of the maximal weight assigned to the prior response, prior evidence and average evidence ($W_R^{\mathrm{max}}$, $W_E^{\mathrm{max}}$ and $W_{CT}^{\mathrm{max}}$, respectively), the uncertainty (*U*) that follows a Beta distribution scaled to the [0,100] range, and the Gaussian distance between current evidence and prior response/evidence/average evidence (the parameter λ controls the relevance of prior information). $W_R^{\mathrm{max}}$, $W_E^{\mathrm{max}}$ and $W_{CT}^{\mathrm{max}}$ allow for attractive and repulsive effects and their absolute values can be compared to quantify which has a larger influence on perception (higher weight). The standard deviation (${\sigma}_{i}$) is defined as a linear model of uncertainty with an intercept ${\alpha}_{\sigma }$ and slope ${\beta}_{\sigma }$. The average evidence up to the current trial (${\overline{E} }_{i}$) is computed through the sum of evidence up to the current trial divided by the number of trials ${N}_{i}$.

## Supplementary Information

Additional file 1: Figures S1-S3. Fig. S1-[Behavioral results versus model predictions per order condition]. Fig. S2-[Posterior distributions of weights assigned to prior information for the three experiments in random order condition]. Fig. S3.-[Central task performance across experiments per participant.]

Additional file 2: Full Model Definition for Average Evidence, Prior Evidence, and Response integration model.

## Data Availability

All data analysed during this study were collected in Experiments 1 to 3 of this study and are publicly available on Open Science Framework, https://osf.io/x4chb [54]. All data generated or analysed during this study are included in this published article, its supplementary information files and publicly available repositories.

## Abbreviations

CI: Credible interval
LOO-CV: Leave-one-out cross validation
PDF: Probability density function
ΔELPD: Difference in expected log-predictive density
