# Supplementary material for: Quantifying effects of inattention on serial dependence in numerosity and orientation judgments using a Bayesian integration approach
Source: BMC Biol. 2026 Oct 31;24:233. doi: 10.1186/s12915-026-02772-8 (PMC13753399; doi:10.1186/s12915-026-02772-8)
Supplement: Supplementary file 1 [file 12915_2026_2772_MOESM1_ESM.pdf]

Supplementary material

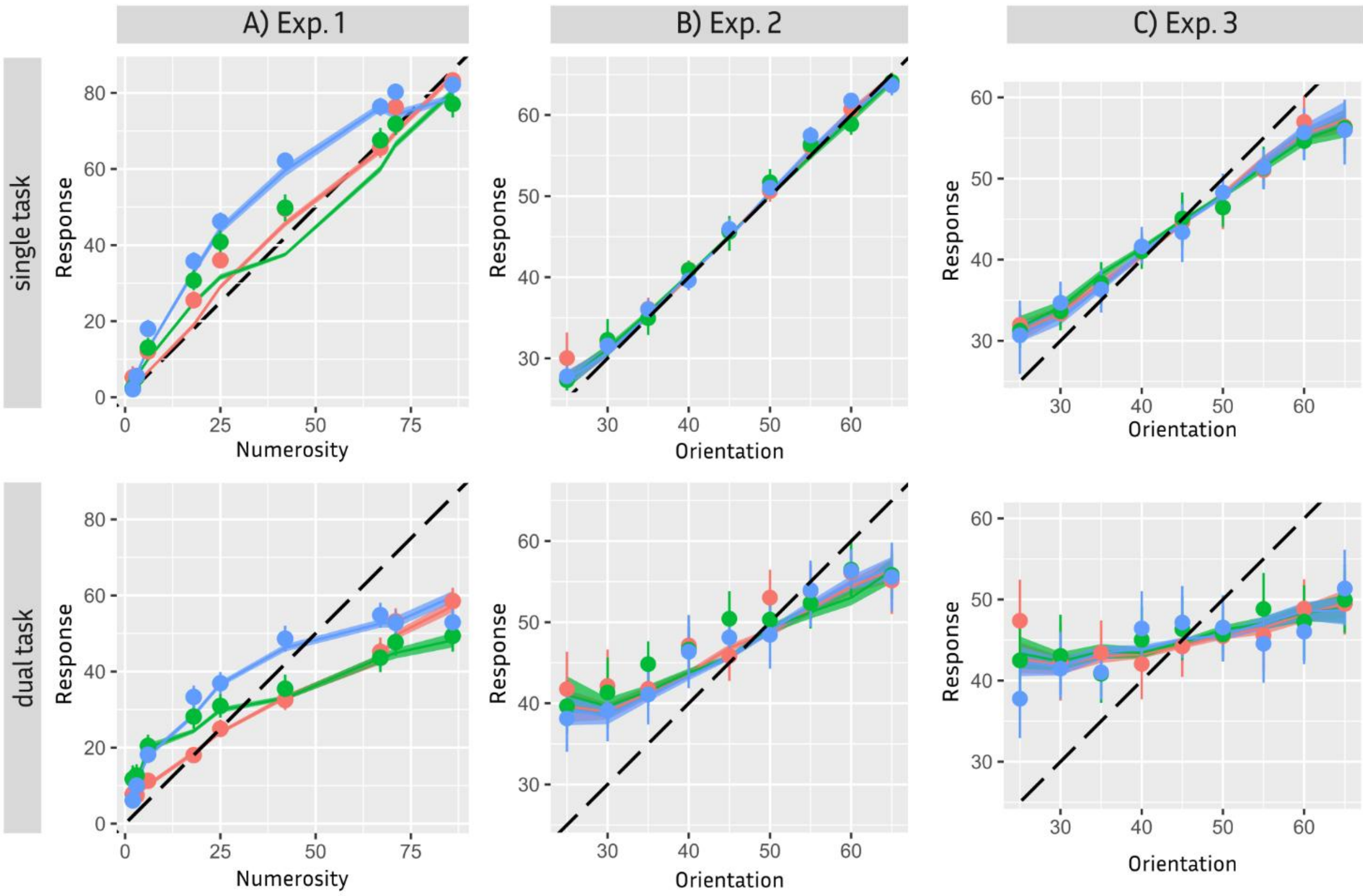

Fig. S1. Behavioral results versus model predictions per order condition. Circles and error bars correspond to the mean and 97% non-parametric bootstrapped confidence intervals for group averages. Stripes depict 97% credible intervals for group average posterior predictions. The rows correspond to the three experiments (A: Experiment 1, B: Experiment 2, C: Experiment 3) and plots illustrate absolute effects with stimulus magnitudes (numerosity or orientation) on the x-axis and responses on the y-axis. The left column shows results for single task and the right column for dual task condition. In all plots, the results are grouped per presentation order condition: descending (red), random (green), and ascending (blue).

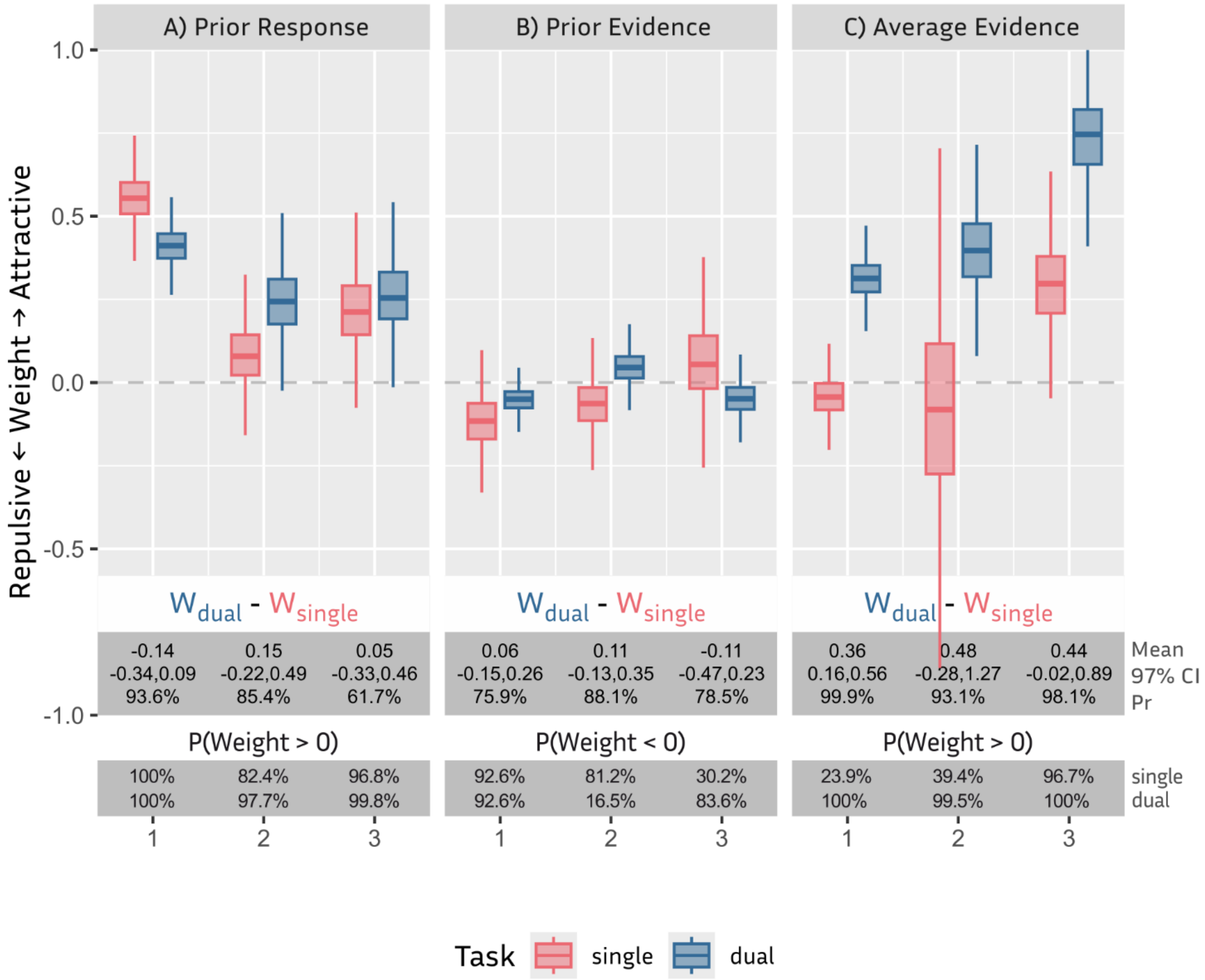

Fig. S2. Posterior distributions of weights assigned to prior information for the three experiments in random order condition. This figure replicates Figure 2 in the main manuscript but includes only trials with random presentation order. The overall effects remained consistent irrespective of the presentation order. Values of the weights are shown on the y-axes. Estimates are grouped per attention condition (single task: red / left; dual task: blue / right). The columns show the weight of A) prior response, B) prior evidence, and C) average evidence, respectively. The inset table shows differences between the two attention conditions (mean and 97% credible interval) and the probability of estimates being higher (positive values in the mean difference) or lower (negative values in the mean difference) in the dual task than the single task condition, and below the proportion of data above/below zero for each weight per experiment and attention condition. Please note that, as in the poor attention (dual task) condition, both prior response and average evidence have an attractive effect, making precise estimation of the corresponding weights problematic, as the model can only account for the total attractive influence of both variables (note the bigger uncertainty about weight estimates for blue boxplots in subplots A and C).

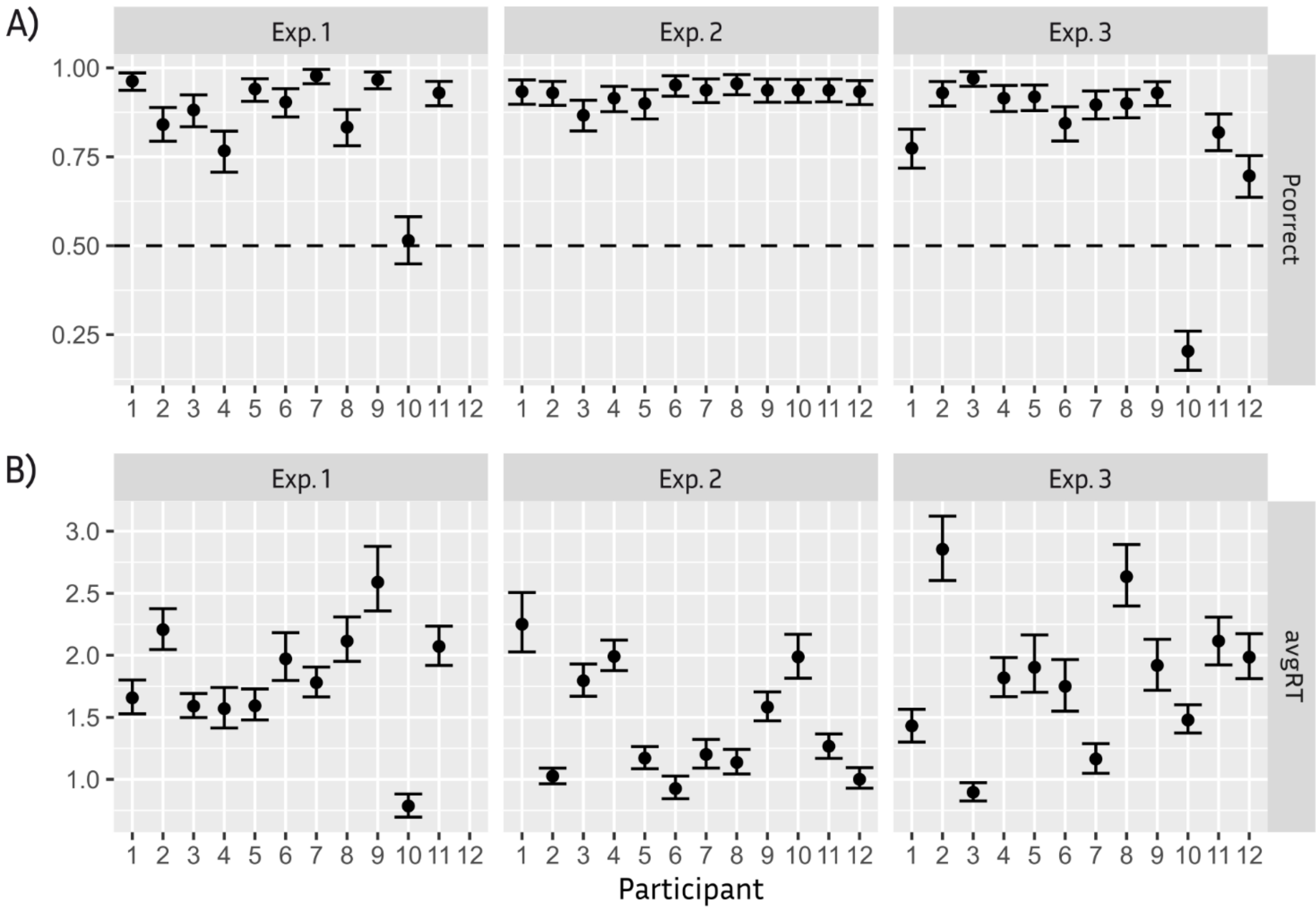

Fig. S3. Central task performance across experiments per participant. Circles and error bars correspond to the mean and 97% non-parametric bootstrapped confidence intervals for participant averages. The columns illustrate central task performance for each experiment respectively. The top row (A) shows the proportion of correctly identified target and non-target stimuli in the central task. This accuracy of the central task can be understood as manipulation check of the dual task condition. The dashed line indicates chance level performance (P(correct) = 50%). In Experiment 1 we excluded participant 10 in our analyses due to their chance level performance in the central task. Please note that we did not exclude participant 10 in Experiment 3 as the participant's accuracy was below chance level. This pattern is consistent with adequate engagement in the central task while suggesting a systematic confusion of the target and non-target response mapping. The bottom row (B) illustrates the average reaction time for the central task per participant.
