# Supplementary material for: Quantifying effects of inattention on serial dependence in numerosity and orientation judgments using a Bayesian integration approach
Source: BMC Biol. 2026 Oct 31;24:233. doi: 10.1186/s12915-026-02772-8 (PMC13753399; doi:10.1186/s12915-026-02772-8)
Supplement: Supplementary file 2 [file 12915_2026_2772_MOESM2_ESM.pdf]

Full Model Definition for Average Evidence, Prior Evidence, and Response integration model

$$R_i \sim Normal(\mu_i, \sigma_i)$$

$$\mu_i = \begin{cases} E_i & i = 1 \\ E_i + W_{CT_{T,P}} \cdot (\bar{E}_i - E_i) + W_{R_{i-1}} \cdot (R_{i-1} - E_i) + W_{E_{i-1}} \cdot (E_{i-1} - E_i) & i > 1 \end{cases}$$

$$\bar{E}_i = \frac{\sum E_i}{N_i}$$

$$W_{R_{i-1}} = W_{R_{T,P}}^{max} U_i e^{-\frac{(E_i - R_{i-1})^2}{\lambda_{T,P}}}$$

$$W_{E_{i-1}} = W_{E_{T,P}}^{max} U_i e^{-\frac{(E_i - R_{i-1})^2}{\lambda_{T,P}}}$$

$$W_{CT} = W_{CT_{T,P}}^{max} U_i e^{-\frac{(E_i - \bar{E}_i)^2}{\lambda_{T,P}}}$$

$$\log(U_i) \sim \frac{BetaP\left(\frac{E_i}{100} \mid U_{max\,T,P}, \kappa_{T,P}\right)}{BetaP\left(U_{max\,T,P} \mid U_{max\,T,P}, \kappa_{T,P}\right)}$$

$$\sigma_i = \alpha_{\sigma_{T,P}} + \beta_{\sigma_{T,P}} U_i$$

$$\begin{bmatrix} logit\left(U_{max_{T=S,P}}\right) \\ \frac{1}{\log\left(\kappa_{T=S,P}\right)} \\ \log\left(\alpha_{\sigma_{T=S,P}}\right) \\ \log\left(\beta_{\sigma_{T=S,P}}\right) \\ logit\left(U_{max_{T=D,P}}\right) \\ \frac{1}{\log\left(\kappa_{T=D,P}\right)} \\ \log\left(\alpha_{\sigma_{T=D,P}}\right) \\ \log\left(\beta_{\sigma_{T=D,P}}\right) \end{bmatrix} \sim MVNormal\left(\begin{bmatrix} \mu_{U\,max_{T=S}} \\ \mu_{\kappa\,T=S} \\ \mu_{\alpha\,\sigma_{T=S}} \\ \mu_{\beta\,\sigma_{T=S}} \\ \mu_{U\,max_{T=D}} \\ \mu_{\kappa\,T=D} \\ \mu_{\alpha\,\sigma_{T=D}} \\ \mu_{\beta\,\sigma_{T=D}} \end{bmatrix}, S_{scale}\right)$$

$$\begin{bmatrix} W_{R_{T=S,P}}^{max} \\ W_{E_{T=S,P}}^{max} \\ W_{CT_{T=S,P}}^{max} \\ 2 * \log\left(\lambda_{T=S,P}\right)^2 \\ W_{R_{T=D,P}}^{max} \\ W_{E_{T=D,P}}^{max} \\ W_{CT_{T=D,P}}^{max} \\ 2 * \log\left(\lambda_{T=D,P}\right)^2 \end{bmatrix} \sim MVNormal\left(\begin{bmatrix} \mu_{W_{R_{T=S}}^{max}} \\ \mu_{W_{E_{T=S}}^{max}} \\ \mu_{W_{CT_{T=S}}^{max}} \\ \mu_{\lambda\,T=S} \\ \mu_{W_{R_{T=D}}^{max}} \\ \mu_{W_{E_{T=D}}^{max}} \\ \mu_{W_{CT_{T=D}}^{max}} \\ \mu_{\lambda\,T=D} \end{bmatrix}, S\right)$$

$$\mu_{U\,max_T} \sim Normal(0, 1)$$

$$\mu_{\kappa_T} \sim Normal(-2.5, 2)$$

$$\mu_{\alpha_{\sigma_T}} \sim Normal(0, 0.5)$$

$$\mu_{\beta_{\sigma_T}} \sim Normal(-2, 1)$$

$$\mu_{W_{R_T}^{max}}, \mu_{W_{E_T}^{max}}, \mu_{W_{CT_T}} \sim Normal(0, 0.5)$$

$$\mu_{\lambda_T} \sim Normal(-0.5, 0.5)$$

$$S_{Scale} = \Sigma R \Sigma$$

$$S = \Sigma R \Sigma$$

$$R \sim \text{LKJcorr}(2)$$

where Σ is a diagonal matrix of variances where each diagonal element $\sigma_i \sim Exponential(1)$.
